# Comprehensive Analysis of mRNA Expression Profiles in Head and Neck Cancer by Using Robust Rank Aggregation and Weighted Gene Coexpression Network Analysis

**DOI:** 10.1155/2020/4908427

**Published:** 2020-12-07

**Authors:** Zaizai Cao, Yinjie Ao, Yu Guo, Shuihong Zhou

**Affiliations:** Department of Otolaryngology, The First Affiliated Hospital, College of Medicine, Zhejiang University, Zhejiang Province 310003, China

## Abstract

**Background:**

Head and neck squamous cell cancer (HNSCC) is the sixth most common cancer in the world; its pathogenic mechanism remains to be further clarified.

**Methods:**

Robust rank aggregation (RRA) analysis was utilized to identify the metasignature dysregulated genes, which were then used for potential drug prediction. Weighted gene coexpression network analysis (WGCNA) was performed on all metasignature genes to find hub genes. DNA methylation analysis, GSEA, functional annotation, and immunocyte infiltration analysis were then performed on hub genes to investigate their potential role in HNSCC.

**Result:**

A total of 862 metasignature genes were identified, and 6 potential drugs were selected based on these genes. Based on the result of WGCNA, six hub genes (*ITM2A*, *GALNTL1*, *FAM107A*, *MFAP4*, *PGM5*, and *OGN*) were selected (GS > 0.1, MM > 0.75, GS *p* value < 0.05, and MM *p* value < 0.05). All six genes were downregulated in tumor tissue (FDR < 0.01) and were related to the clinical stage and prognosis of HNSCC in different degrees. Methylation analysis showed that the dysregulation of *ITM2A*, *GALNTL1*, *FAM107A*, and *MFAP4* may be caused by hypermethylation. Moreover, the expression level of all 6 hub genes was positively associated with immune cell infiltration, and the result of GSEA showed that all hub genes may be involved in the process of immunoregulation.

**Conclusion:**

All identified hub genes could be potential biomarkers for HNSCC and provide a new insight into the diagnosis and treatment of head and neck tumors.

## 1. Introduction

Head and neck squamous cell carcinoma (HNSC) is the sixth most common cancer in the world [1]. Worldwide, more than 300000 patients die of HNSC every year [2]. Although many treatments for HNSC such as surgery, chemotherapy, and radiotherapy have obtained some success, the 5-year survival rate is still only 40-50% [3]. The chances of survival for patients with HNSCC depend largely on the initial stage of cancer. Therefore, early detection and accurate diagnosis are crucial for patients with HNSCC to receive treatment.

In the past twenty years, with the application of microarray and next-generation sequencing technologies, a great number of novel diagnostic or therapeutic biomarkers have been identified in HNSCC [4]. However, small samples in independent research, different platform technologies, and different screening criteria have a great impact on the research results. To solve this problem and obtain stable biomarkers, researchers proposed a novel rank aggregation method: robust rank aggregation (RRA) [5], which has been implemented as an R package (RobustRankAggreg) [5], to identify the overlapping genes among ranked gene lists [6], thus making the result more reliable.

WGCNA [7] is an effective method to find the clusters of highly correlated genes and identify the hub genes of each cluster [7]. This method has been widely applied in various biological contexts. In our study, a total of 24 independent gene datasets were included in RRA analysis to identify robust DEGs. We used these DEGs to predict the potential small molecular drugs. The coexpression network was then established by WGCNA to identify hub genes in these robust DEGs. The role of all hub genes in HNSCC was then validated by other independent databases. Furthermore, we also utilized multiple online tools such as DiseaseMeth [8], MEXPRESS [9], and MethSurv [10] to evaluate the methylation level of hub genes. TIMER was used to assess the association between immune infiltration and hub genes. GSEA [11] analysis was applied to explore the biological functions of these hub genes. To the best of our knowledge, this is the first time to utilize RRA and WGCNA simultaneously for screening biomarkers of HNSCC.

## 2. Result

### 2.1. Metasignature DEGs Identified by RRA Analysis

**Figure 1:**
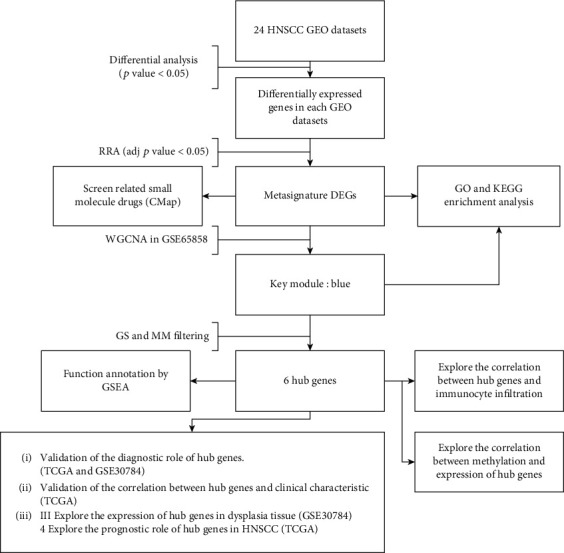
Simple flow chart of the entire study.

**Figure 2:**
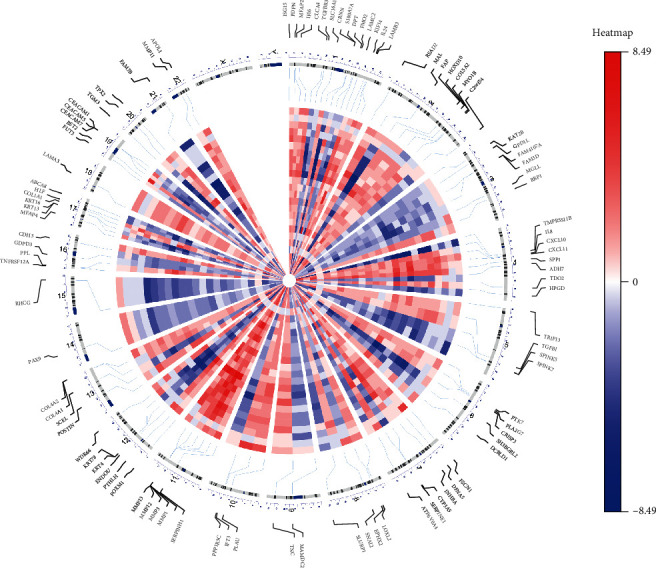
Circular visualization of expression patterns and chromosomal positions of top 100 DEGs. Red indicates gene upregulation, blue indicates downregulation, and white indicates genes that do not exist in a given dataset.

The workflow of our study is shown in Figure 1, 24 independent studies were used in RRA analysis, and a total of 466 upregulated genes and 396 downregulated genes were identified. The top 5 upregulated genes in tumor tissue were *MMP1* (FDR = 4.77*e* − 53), *MMP10* (FDR = 8.25*e* − 40), *PTHLH* (FDR = 1.48*e* − 38), *MMP3* (FDR = 5.38*e* − 38), and *SPP1* (FDR = 2.79*e* − 37) while *TMPRSS11B* (FDR = 1.53*e* − 36), *MAL* (FDR = 8.50*e* − 35), *CRISP3* (FDR = 1.60*e* − 34), *CRNN* (FDR = 4.32*e* − 33), and *KRT4* (FDR = 1.24*e* − 30) were the most significant downregulated genes. The chromosomal locations and expression patterns of the top 100 DEGs are visualized in Figure 2. Chromosome 1 contains most metasignature DEGs while X and Y chromosome contains no DEGs. It is clear that almost all displayed genes have the same expression pattern in most of the independent studies, which indicates the reliability of the RRA analysis result.

### 2.2. Functional Enrichment Analysis

**Figure 3:**
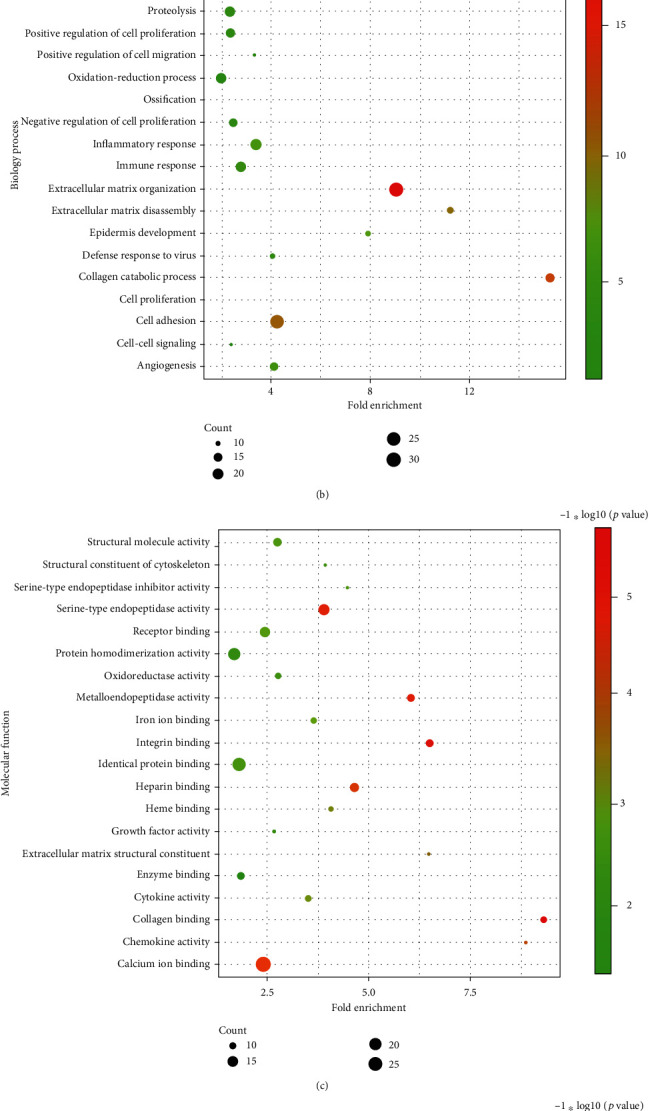
GO and KEGG analyses of the top 300 DEGs. (a) The correlation between genes and KEGG pathway. (b) The correlation between genes and GO terms of biological process. (c) The correlation between genes and GO terms of molecular function. (d) The correlation between genes and GO terms of cellular component.

We select the top 300 DEGs to perform GO and KEGG enrichment analyses. Among the KEGG pathway database, we can find that these DEGs were enriched in multiple cancer-related pathways like focal adhesion, PI3K-Akt signal pathway, pathway in cancer, small-cell lung cancer, transcriptional misregulation in cancer, and chemical carcinogenesis (Figure 3(a)). Furthermore, in all terms of KEGG and GO, we found that these metasignature genes mostly involved in pathways associated with the construction of ECM such as ECM receptor interaction, extracellular matrix organization, collagen catabolic process, collagen binding, collagen trimer, extracellular region, and extracellular exosome (Figure 3).

### 2.3. Screen the Candidate Small Molecule Drugs for HNSCC

According to our screen criteria, 6 small molecule drugs (repaglinide ES = −0.848, thiostrepton ES = −0.863, levamisole ES = −0.75, cortisone ES = −0.866, zimeldine ES = −0.784, and cyproterone ES = −0.742) were identified (Table 1). Their 2D structures are visualized in Supplementary Fig 1. These potential drugs can to some extent reverse the robust dysregulated genes in HNSCC, thus providing suggestions for us to develop targeted drugs.

### 2.4. Identification of Hub Genes in HNSCC Patients

**Figure 4:**
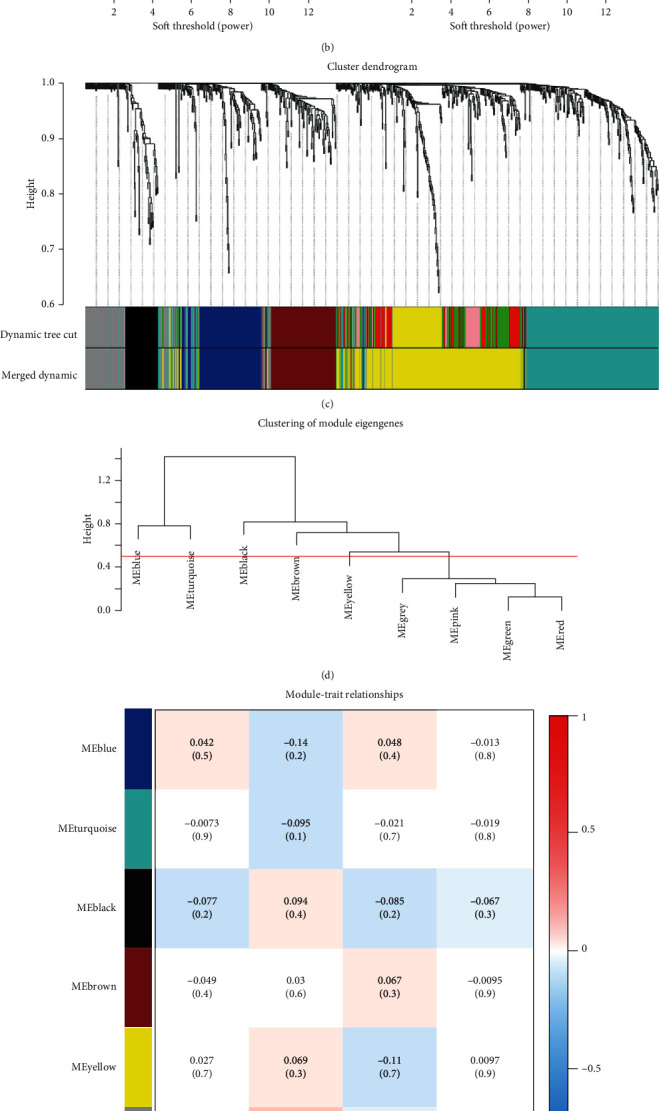
Identification of hub genes in HNSCC by using WGCNA. (a) Clustering dendrograms of genes. (b) Analysis of the scale-free fit index and the mean connectivity for various soft-thresholding powers. (c) Dendrogram of all DEGs clustered based on a dissimilarity measure. (d) Clustering of modules. (e) Heatmap of the correlation between modules and clinical traits. (f) Correlation matrix of each hub genes.

To identify the hub genes, we performed WGCNA on the GSE65858, which included 270 samples from HNSCC patients with complete clinical data. Six different gene modules were identified (Figure 4) according to the result of cluster analysis on expression data of metasignature DEGs. The correlation coefficients between each module and each clinical trait were calculated, and it is clear that only the blue module and gray module were significantly associated with T grade of HNSCC (Figure 4(e)). Because genes in the gray module are not significantly coexpressed with each other, we only chose the blue module as a key module. A total of 102 genes were included in blue modules, and the result of enrichment analysis for these genes showed that the most significant GO and KEGG terms were related to cell metabolism, chemokine activity, and transmembrane transport (Supplementary Fig 2). According to the value of GS and MM (GS > 0.1, MM > 0.75, GS *p* value < 0.05, and MM *p* value < 0.05), 6 genes (*ITM2A*, *GALNTL1*, *OGN*, *FAM107A*, *MFAP4*, and *PGM5*), which were also significantly correlated with each other (Figure 4(f)), were selected from the blue module.

### 2.5. Validate the Role of Hub Genes in HNSCC

**Figure 5:**
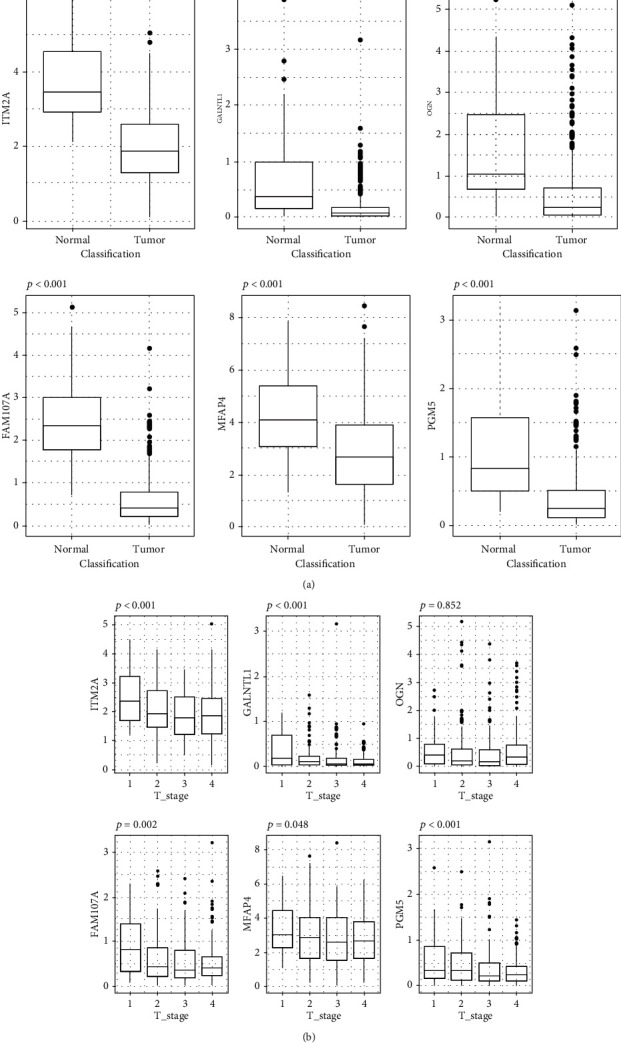
Validation of hub genes in the TCGA database. (a) The correlation between hub genes and sample type. (b) The correlation between hub genes and tumor T stage.

To further validate the diagnostic role of hub genes, we compare the expression level of these genes between normal tissue and tumor tissue in the TCGA database. Considering the result of WGCNA which revealed a negative association between hub genes and tumor T grade, we also used the TCGA database to validate the role of hub genes in TN grade of HNSCC. In Figure 5(a), it is clear that all 6 hub genes were remarkably different between normal and tumor tissues, and the ROC curve indicates a high diagnostic value of all hub genes (Supplementary Fig 4A). In Figure 5(b), we can see that five hub genes (*ITM2A*, *GALNTL1*, *FAM107A*, *MFAP4*, and *PGM5*) were upregulated in the T1-T2 stage and downregulated in the T3-T4 stage, which is consistent with the result in WGCNA. However, there is no correlation between hub genes and tumor N stage (Supplementary Fig 3). The result above indicated that these hub genes may affect the growth rather than metastasis of the tumor.

### 2.6. Explore the Role of Hub Genes in Malignant Transformation and Prognosis

**Figure 6:**
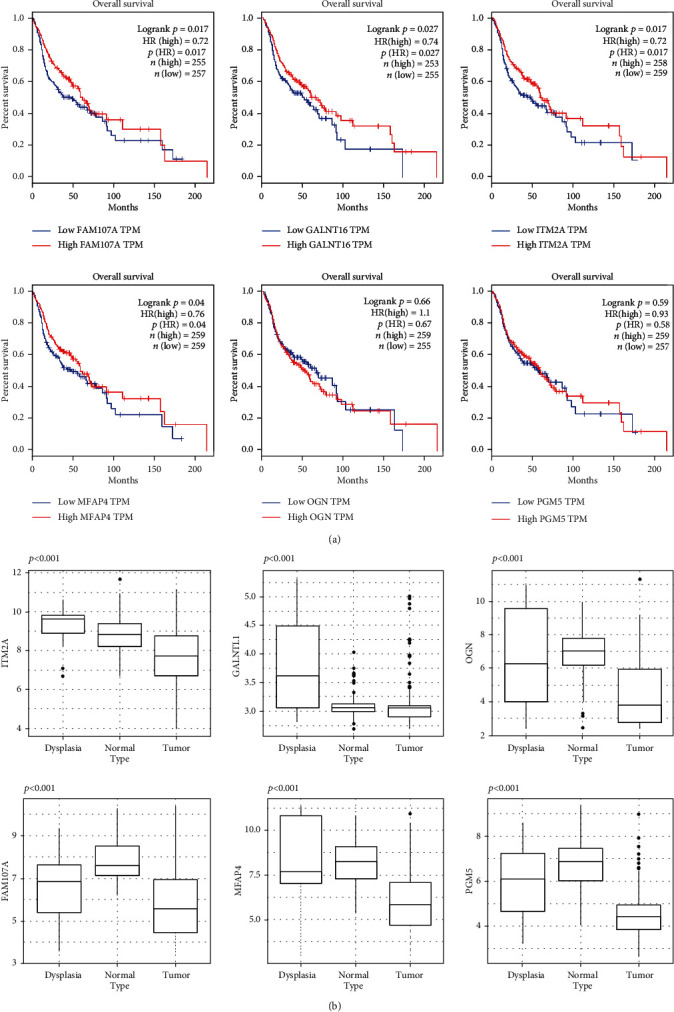
The role of hub genes in malignant transformation and prognosis: (a) prognostic role of all hub genes; (b) the expression level of hub genes in normal, dysplasia, and tumor tissues.

GSE30748 provides the gene expression data of oral dysplasia tissue. Compared with tumor tissue, we found that all hub genes were significantly higher expressed in dysplasia tissue (Figure 6(b)); except for *FAM107A* with AUC = 0.66, the other 5 hub genes have AUC > 0.7 (Supplementary Fig 4B). We also explore the prognostic role of all these hub genes by using the GEPIA website [12]. The KM curve showed that the lower expression of four hub genes (*ITM2A*HR = 0.72, *GALNTL1*HR = 0.74, *FAM107A*HR = 0.72, and *MFAP4*HR = 0.76) was significantly associated with poor overall survival (Figure 6(a)).

### 2.7. DNA Methylation and Expression of Hub Genes

**Figure 7:**
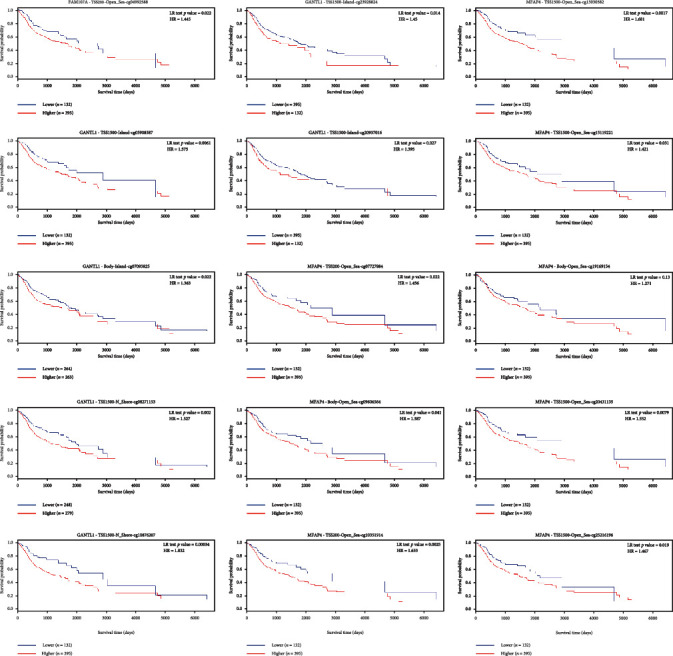
15 methylation sites of hub genes with prognostic ability in HNSCC.

As we all know, methylation can significantly affect the expression of multiple genes; therefore, we at first used DiseaseMeth 2.0 to explore the mean methylation level of hub genes. Because *OGN* was not included in DiseaseMeth, we only explore the other 5 genes. We found that the mean methylation level of *ITM2A*, *GALNTL1*, *FAM107A*, and *MFAP4* was significantly higher in tumor tissue while the methylation level of PGM5 was higher in normal tissue (Supplementary Fig 5). This indicates that the low expression PGM5 in HNSCC may not be caused by methylation. We next explore the relationship between four hub genes and their methylation site. From Supplementary Fig 6, we can see that various methylation sites on each gene were negatively correlated with the expression level of the corresponding gene, indicating that downregulation of four hub genes (*ITM2A*, *GALNTL1*, *FAM107A*, and *MFAP4*) may be caused by hypermethylation. To find the key methylation site of hub genes, we also used MethSurv to explore the prognostic role of these methylation sites (*r* < 0 and adjusted *p* value < 0.05). A total of 15 methylation sites were found to be important prognostic factors for HNSCC (Figure 7).

### 2.8. Immune Infiltration and Hub Genes

**Figure 8:**
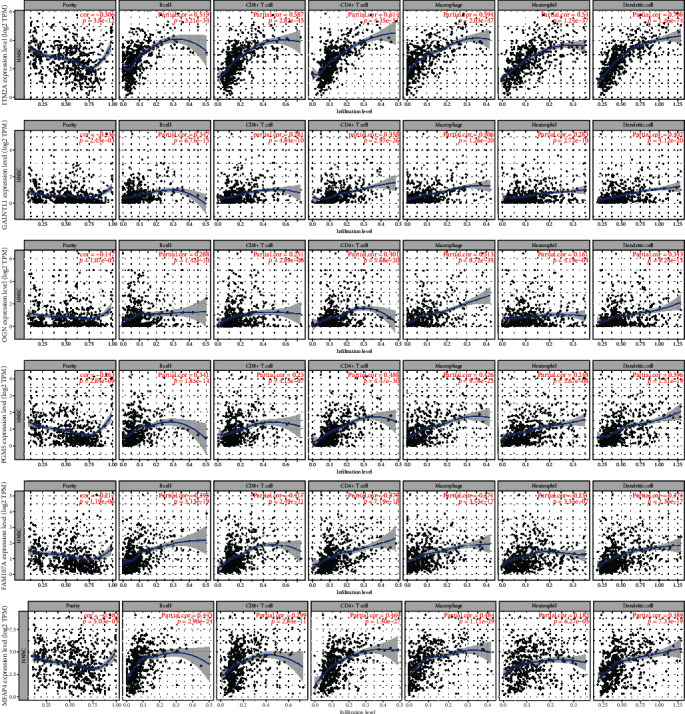
The correlation between hub genes and infiltration of immune cells.

The tumor microenvironment comprises multiple kinds of cells such as epithelial cells, fibroblasts, and immune cells. A great number of studies have revealed the significant role of immune cells in various cancers. Therefore, we used TIMER to investigate the association between hub genes and different kinds of cells. It is interesting that we found that all hub genes were negatively correlated with tumor purity. On the contrary, 6 hub genes were all positively related to the infiltration of immune cells (Figure 8).

### 2.9. GSEA Revealed Pathway Dysregulated by Hub Genes

To further explore the expression pathway of all 6 hub genes, GSEA analysis was performed for each gene. Supplementary Fig 7 represents the top 10 enriched pathways in each hub gene (ranked by enrichment score). According to the result of GSEA, we found that multiple immune-related pathways were significantly enriched in the higher expression group of hub genes like allograft rejection, primary immunodeficiency, intestinal immune network for IgA production, T cell receptor signaling pathway, B cell receptor signaling pathway, autoimmune thyroid disease, graft-versus-host disease, human T cell leukemia virus 1 infection, leukocyte transendothelial migration, Th1 and Th2 cell differentiation, Th17 cell differentiation, and asthma.

## 3. Discussion

To identify the robust dysregulated genes in HNSCC, we included a total of 24 independent datasets for RRA analysis. A total of 466 upregulated genes and 396 downregulated genes were identified. The top 5 upregulated genes mostly came from matrix metalloproteinase (MMP) families. Its family members have been proved to play a vital role in the progression, invasion, and metastasis of HNSCC [13]. The most downregulated gene is *TMPRSS11B*, a member of the type II transmembrane serine protease family. It has been reported to be downregulated in multiple epithelial cancers [14]. To further understand the biological function of these metasignature genes, we performed GO and KEGG analyses on the top 300 metasignature DEGs. Multiple cancer-related pathways such as transcriptional misregulation, PI3K-Akt signaling pathway, pathways in cancer, and ECM receptor interaction were significantly enriched, confirming the important role of these DEGs in HNSCC. Furthermore, many enriched terms were associated with the construction of ECM, indicating the importance of the microenvironment in the development of HNSCC. According to the results of enrichment analyses, we confirmed that these metasignature DEGs are significantly related to the occurrence and development of HNSCC.

After identifying the robust DEGs in HNSCC, we try to use the expression pattern of these genes to predict the potential small molecule drugs. The CMap database was used, and six small molecule drugs were selected because they can reverse the expression pattern of metasignature DEGs. Among all these drugs, four of which have been studied in HNSCC previously. For instance, thiostrepton has been reported to affect the proliferation, apoptosis, and radiosensitivity in head and neck cancer [15, 16]. Levamisole also has been used in HNSCC before, but its effect is still controversial [17]. Cyproterone and cortisone are both hormone medicines. However, there is no strong evidence that hormone therapy is effective for head and neck tumors. Repaglinide is a hypoglycemic agent while zimeldine is a kind of antidepressant drug, both of which have not been studied as a drug for HNSCC. Considering that the mortality rate of head and neck tumors has not improved significantly in the past ten years, traditional treatment methods like surgery and radiotherapy may not be enough for HNSCC; it is meaningful to further reveal the potential of chemical molecules in targeted therapy of HNSCC.

To identify the hub genes among all 862 metasignature DEGs, WGCNA was utilized to construct a coexpression network. Finally, we identified 6 hub genes (*ITM2A*, *GALNTL1*, *OGN*, *FAM107A*, *MFAP4*, and *PGM5*) according to our selection criteria. We used other independent databases to validate the expression pattern and clinical relationship of these hub genes. The result showed that all hub genes were downregulated in tumor tissue and were negatively correlated with tumor T stage. Furthermore, compared with tumor tissue, these 6 hub genes were also downregulated in dysplasia tissue. The ROC curve indicated that these genes may help us better identify the HNSCC. Besides, four genes (*ITM2A*, *GALNTL1*, *FAM107A*, and *MFAP4*) also performed well in the prognosis prediction of HNSCC. Interestingly, all 6 hub genes were seldom explored in HNSCC previously. *ITM2A*, a family member of BRICHOS, has been reported to be downregulated in both breast and ovarian cancers which may affect the proliferation and autophagy process of tumors [18, 19]. However, its role in HNSCC has not been fully studied. Similarly, the role of another 5 hub genes in cancer also has been reported previously to varying degrees. For example, *PGM5* was identified as a diagnostic and prognostic biomarker in liver and colorectal cancers [20, 21]. The higher expression of antisense chain of *PGM5* was showed to inhibit the proliferation and metastasis of tumors [22]. The higher expression of *OGN* was also reported to inhibit the process of EMT through the EGFR/Akt pathway [23]. However, the role of these genes in the development of HNSCC remains unclear.

As we all know, hypermethylation is an important cause of the downregulation of gene expression. A recent study showed that hypermethylation may lead to the low expression of *FAM107A* in laryngeal cancer [24], which is consistent with our results. Through methylation analysis, we also find that the low expression of another three hub genes (*ITM2A*, *GALNTL1*, and *MFAP4*) may be significantly associated with hypermethylation in multiple methylation sites. Because DNA methylation is a reversible process, targeted therapies for the unique methylation site of the tumor are promising. To further screen out methylation sites with research potential, we also performed survival analysis and found that hypermethylation of 15 methylation sites in *FAM107A*, *GALNTL1*, and *MFAP4* was significantly associated with poor overall survival. All selected hub genes and their methylation conditions may help us better judge the state of HNSCC (inert or invasive), so as to develop a more appropriate treatment strategy.

A great number of previous researches have revealed that the infiltration of immune cells in the tumor microenvironment could largely affect the development of cancer cells [25, 26]. Therefore, we used TIMER to explore the relationship between hub genes and immune cell infiltration. Interestingly, all six hub genes were positively correlated with infiltration of B cell, CD8+ T cell, CD4+ T cell, macrophage, neutrophil, and dendritic cells, indicating that our hub genes may to some extent play a role in immunological regulation. The results of GSEA further support this hypothesis; a great number of immune-related pathways were significantly enriched in higher expression groups of hub genes. A recent study confirmed our result; Hu et al. point out that higher expression of *OGN* can promote the infiltration of CD8+ T cells thus inhibiting the formation of new blood vessels in colorectal cancer [27]. Some studies also have described the role of some hub genes (*ITM2A*, *MFAP4*) in immunoregulation [28, 29]. However, the role of these genes in tumor immune regulation is still not fully illustrated; we need more experiments to validate the association between these hub genes and immune infiltration.

## 4. Conclusion

In conclusion, by utilizing the RRA method, we identified a series of robust DEGs in HNSCC. Based on WGCNA, 6 hub genes (*ITM2A*, *GALNTL1*, *OGN*, *FAM107A*, *MFAP4*, and *PGM5*) in the blue module were selected. All hub genes were significantly downregulated in tumor tissue of HNSCC. The expression pattern of four hub genes (*ITM2A*, *GALNTL1*, *FAM107A*, and *MFAP4*) may be caused by hypermethylation. All six hub genes may play a role in immunological regulation in the microenvironment of HNSCC which need more experiment to verify.

## 5. Materials and Method

### 5.1. Selection of Included Datasets

The mRNA expression profile-related datasets were searched in the GEO database by using the keywords head and neck cancer, larynx, laryngeal, tongue, mouth, oral, oropharynx, tonsil, hypopharynx, and hard palate. Two people independently screened the datasets based on the inclusion criteria as follows: (1) Included datasets must provide the gene expression profile of HNSCC and corresponding normal tissue control. (2) Each group of one dataset should contain at least 5 samples. (3) The platform of each study should contain more than 8000 genes. Finally, a total of 25 studies were included in our research, and among which, 24 independent studies were used for RRA analysis; one dataset (GSE30784) with gene expression data of dysplasia tissue was used for further validation and exploration. Detailed information of included GEO datasets is shown in Table 2.

### 5.2. Identification of Robust DEGs

R package “GEOquery” was used to directly obtain series matrix files, sample phenotype data, and corresponding platform information from the GEO database. We used “limma” R package to normalize the data and obtain DEGs of each study (*p* value < 0.05). The up- or downregulated genes were arranged from large to small according to the absolute value of logFC. The “RobustRankAggreg” package in R was created for comparison of ranked gene lists and identification of metasignature genes. The result of RRA can help us identify more robust genes from different studies, and the detailed method of the RRA method has been described by previous articles [30]. In the end, the *p* value of the output result was subjected to Bonferroni correction, and mRNA with adjusted *p* value < 0.05 was considered significantly dysregulated. Furthermore, “OmicCircos” R package was utilized to visualize the expression patterns of the top 100 metasignature DEGs in each included study (dysregulated genes according to adjusted *p* value).

### 5.3. Enrichment Analysis

We used DAVID Bioinformatics Resources 6.8 (DAVID; http://david.abcc.ncifcrf.gov/) to annotate the top 300 metasignature genes. GO and KEGG enrichment analyses were performed by using the prediction tool on the website. Bubble charts were used to visualize the top 20 terms of enrichment results.

### 5.4. Identification of Potential Drug for HNSCC

The Connectivity Map (CMap) [31] database (http://www.broadinstitute.org) can help us to predict the potential drugs which can reverse the expression of specific genes. In this study, we input the top 300 metasignature genes (165 upregulated and 135 downregulated genes) into the online tool of CMap for gene set enrichment analysis. Each small molecule will be assigned an enrichment score between -1 and 1. The lower the enrichment score, the better the drug effect to reverse the state of HNSCC cells. In our study, drugs with *p* value < 0.01 and the enrichment score < −0.7 were considered potential small molecules. We also used PubChem (http://www.pubchem.ncbi.nlm.gov) to visualize the 2D structure of selected small molecules.

### 5.5. Key Module and Hub Genes Identified by WGCNA

A total of 862 metasignature genes were included for WGCNA with expression data from GSE65858. We construct a gene coexpression network for all metasignature DEGs; “WGCNA” R package was applied to explore the relationship between each coexpression module and clinical phenotype. A correlation matrix was constructed which was subsequently transformed to a TOM matrix based on the soft threshold (*β* = 7, *R*^2^ = 0.9). All metasignature genes were distributed in different gene modules according to the value of the TOM matrix. Here, we set the minimal module size as 15 and cut height as 0.5. The module with a significant correlation with clinical characteristics was selected. GO and KEGG enrichment analyses were performed on the clinical-related modules. We selected the hub gene according to the value of GS and MM (GS > 0.1, MM > 0.75, GS *p* value < 0.05, and MM *p* value < 0.05).

### 5.6. Verify the Clinical Relevance of Hub Genes

We used the TCGA database at first to validate the diagnostic role of hub genes and the relationship between hub genes and clinical characteristics. We also used an independent dataset (GSE30748) to explore the hub genes' expression levels between dysplasia tissue and tumor tissue. The Student *t*-test or one-way analysis of variance (ANOVA) was used appropriately to test the result of the comparison. Furthermore, we also plot the ROC curves to assess hub genes' diagnostic value; the area under the ROC curve (AUC) was calculated by the “pROC” R package. Survival analysis was also performed on all hub genes by using GEPIA (a visualization website based on the TCGA database: http://gepia.cancer-pku.cn/). The median is considered to be the cutoff for high and low expression of hub genes.

### 5.7. Methylation Analysis

In order to further explore the reason for the dysregulation of hub gens, we performed methylation analysis on all hub genes based on DiseaseMeth 2.0 [8] (http://bioinfo.hrbmu.edu.cn/diseasemeth/), which is a website focusing on collecting methylation data from various tumor tissue. We compare the mean value of methylation between HNSCC and corresponding normal tissue. Furthermore, we also used MEXPRESS [9] (http://mexpress.be) to explore the association between the expression level of hub genes and the methylation level of the corresponding methylation site. Those methylation sites that are negatively correlated with gene expression are defined as candidate sites. To further screen potential key methylation sites, we also conducted survival analyses on these candidate sites by using MethSurv [10] (https://biit.cs.ut.ee/methsurv/).

### 5.8. Immune Cell Infiltration and Hub Genes

To explore the association between immune cell infiltration and expression level of hub genes, we used TIMER [32] (https://cistrome.shinyapps.io/timer/), an online tool based on the TCGA database, to evaluate the infiltration score for six kinds of important immune cells (B cells, CD4+ T cells, CD8+ T cells, neutrophils, macrophages, and dendritic cells). The Pearson correlation coefficient between hub genes and the infiltration score were then calculated.

### 5.9. Gene Set Enrichment Analysis

According to the mean expression value of 6 hub genes, all HNSCC samples in the TCGA database were divided into high expression groups and low expression groups. GSEA analysis was performed and visualized by using the “clusterprofiler” R package. The KEGG gene set was directly downloaded from MSigDB (http://software.broadinstitute.org/gsea/msigdb/index.jsp).

## Figures and Tables

**Table 1:** Six candidate small molecule drugs.

Drug name	Mean value of correlation coefficient	Number of experiments	Enrichment score	*p* value
Repaglinide	-0.685	4	-0.848	0.00097
Thiostrepton	-0.659	4	-0.863	0.00064
Levamisole	-0.619	4	-0.75	0.00784
Cortisone	-0.606	3	-0.866	0.00479
Zimeldine	-0.575	5	-0.784	0.00086
Cyproterone	-0.552	4	-0.742	0.00875

**Table 2:** Detailed information of 24 datasets which were used in RRA analysis.

Time	Gene set	Platform	Number of probes	Country	Tumor type	Tumor sample number	Control sample number
2008	GSE10121	GPL6353	33484	Germany	OSCC	35	6
2017	GSE103412	GPL23978	39321	Denmark	OSCC	23	9
2008	GSE13399	GPL7540	36197	USA	HNSCC	8	8
2008	GSE13601	GPL8300	12625	USA	OSCC	31	26
2019	GSE138206	GPL570	9442	China	OSCC	6	6
2020	GSE143224	GPL5175	19076	Brazil	LSCC	14	11
2010	GSE23036	GPL571	22277	Spain	HNSCC	63	5
2010	GSE23558	GPL6480	41000	India	OSCC	27	5
2010	GSE25099	GPL5175	17881	China	OSCC	57	22
2011	GSE29330	GPL570	54675	USA	HNSCC	13	5
2011	GSE31056	GPL10526	17788	USA	OSCC	23	73
2011	GSE33205	GPL5175	22011	USA	HNSCC	44	25
2011	GSE34105	GPL14951	29377	Sweden	OSCC	62	16
2012	GSE37991	GPL6883	24526	China	OSCC	40	40
2012	GSE42743	GPL570	54645	USA	OSCC	74	29
2013	GSE51985	GPL10558	47220	China	LSCC	10	10
2014	GSE55550	GPL17077	50739	USA	OSCC	139	16
2014	GSE58911	GPL6244	33297	USA	HNSCC	15	15
2014	GSE59102	GPL6480	34664	Brazil	LSCC	29	13
2007	GSE6631	GPL8300	12625	USA	HNSCC	22	22
2007	GSE6791	GPL570	54675	USA	HNSCC	42	14
2016	GSE83519	GPL4133	43376	Netherlands	HNSCC	22	22
2016	GSE84957	GPL17843	77162	China	LSCC	9	9
2007	GSE9844	GPL570	54645	USA	OSCC	26	12

LSCC: laryngeal squamous cell cancer; OSCC: oral squamous cell cancer; HNSCC: head and neck cancer.

## Data Availability

Our manuscript report is the secondary analysis of data from a public database; all data used in the manuscript were mainly from GEO and TCGA databases.

## Abbreviations

RRA: Robust rank aggregation
WGCNA: Weighted gene coexpression network analysis
HNSCC: Head and neck squamous cancer
DEGs: Differentially expressed genes
GO: Gene Ontology
KEGG: Kyoto Encyclopedia of Genes and Genomes
BP: Biology process
MF: Molecular function
CC: Cell components
ECM: Extracellular matrix
TOM: Topological overlap matrix
GS: Gene significance
MM: Module membership
GEPIA: Gene expression profiling interactive analysis
KM curve: Kaplan-Meier curve
EMT: Epithelial to mesenchymal transition
MSigDB: Molecular signature database
TIMER: Tumor immune estimation resource
GSEA: Gene set variation analysis.

## Supplementary Materials

Supplementary Materials Supplementary Fig 1: 2D molecular structure of potential drugs: A: thiostrepton, B: cortisone, C: cyproterone, D: levamisole, E: zimeldine, and F: repaglinide. Supplementary Fig 2: GO and KEGG analyses of blue module: A: the correlation between the blue module and KEGG pathway. B: the correlation between blue module and GO terms of biological process. C: the correlation between blue module and GO terms of molecular function. D: the correlation between blue module and GO terms of cellular component. Supplementary Fig 3: the correlation between hub genes and tumor N stage. Supplementary Fig 4: ROC curves validate the diagnostic role of hub genes. A: diagnostic role of hub genes between normal tissue and tumor tissue. B: diagnostic role of hub genes between tumor tissue and dysplasia tissue. Supplementary Fig 5: mean methylation level of hub genes between normal and tumor tissues. Supplementary Fig 6: the relationship between four hub genes and their methylation site. Supplementary Fig 7: GSEA result of hub genes.
