# Supplementary material for: Comprehensive Analysis of mRNA Expression Profiles in Head and Neck Cancer by Using Robust Rank Aggregation and Weighted Gene Coexpression Network Analysis
Source: Biomed Res Int. 2020 Dec 7;2020:4908427. doi: 10.1155/2020/4908427 (PMC7746451; doi:10.1155/2020/4908427)
Supplement: Supplementary Materials — Supplementary Fig 1: 2D molecular structure of potential drugs: A: thiostrepton, B: cortisone, C: cyproterone, D: levamisole, E: zimeldine, and F: repaglinide. Supplementary Fig 2: GO and KEGG analyses of blue module: A: the correlation between the blue module and KEGG pathway. B: the correlation between blue module and GO terms of biological process. C: the correlation between blue module and GO terms of molecular function. D: the correlation between blue module and GO terms of cellular component. Supplementary Fig 3: the correlation between hub genes and tumor N stage. Supplementary Fig 4: ROC curves validate the diagnostic role of hub genes. A: diagnostic role of hub genes between normal tissue and tumor tissue. B: diagnostic role of hub genes between tumor tissue and dysplasia tissue. Supplementary Fig 5: mean methylation level of hub genes between normal and tumor tissues. Supplementary Fig 6: the relationship between four hub genes and their methylation site. Supplementary Fig 7: GSEA result of hub genes. [file 4908427.f1.pdf]

Supplementary Fig 1 2D molecular Structure of potential drugs. A: thiostrepton B: cortisone C: cyproterone D: levamisole E: zimeldine F: repaglinide

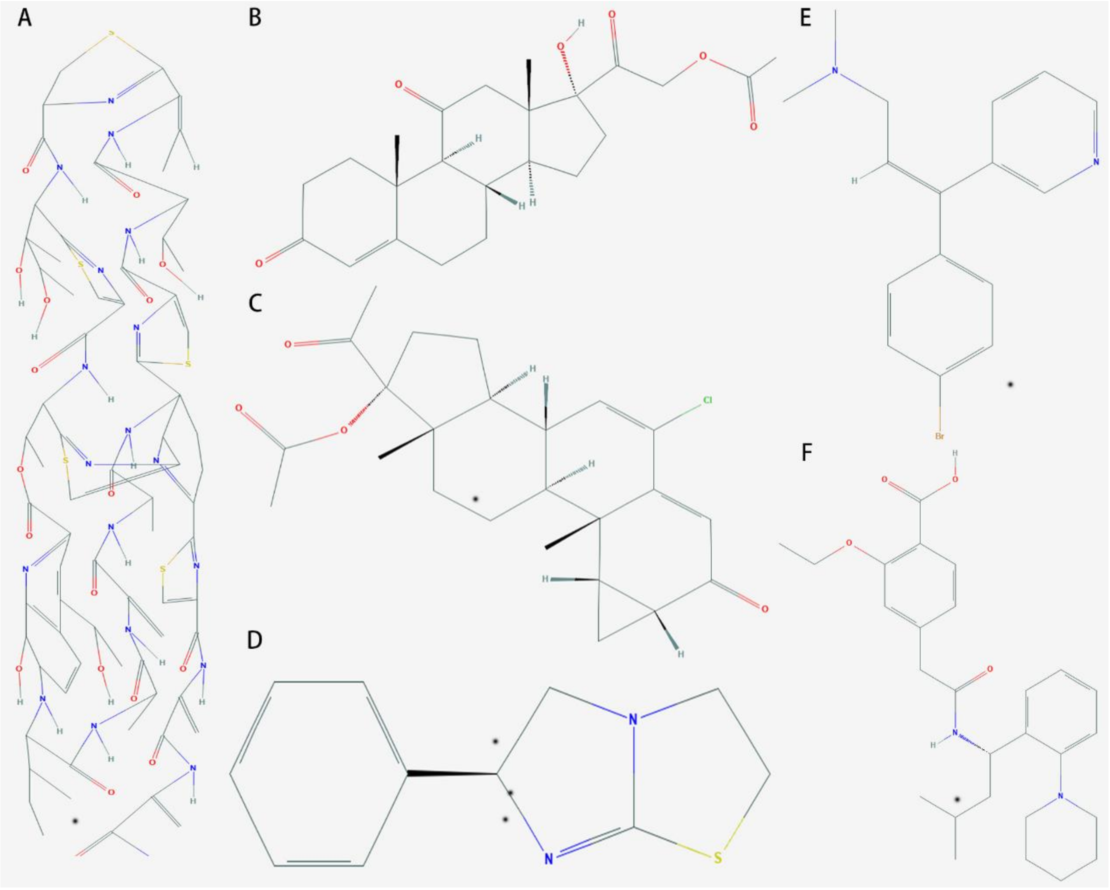

Supplementary Fig 2 GO and KEGG analysis of blue module. A: The correlation between blue module and KEGG pathway. B: The correlation between blue module and GO terms of biological process. C: The correlation between blue module and GO terms of molecular function. D: The correlation between blue module and GO terms of cellular component.

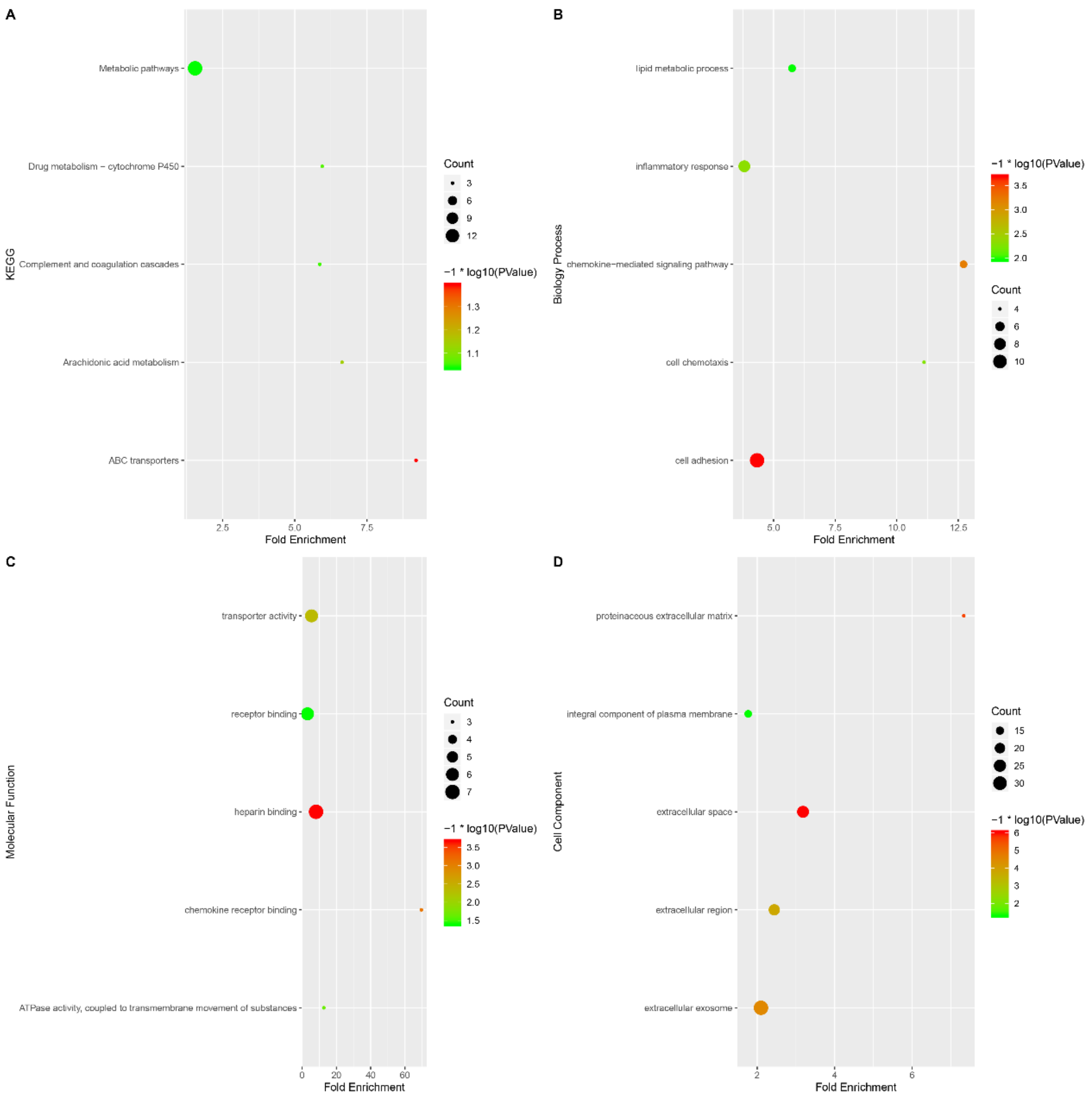
A
KEGG
Metabolic pathways
Drug metabolism − cytochrome P450
Complement and coagulation cascades
Arachidonic acid metabolism
ABC transporters
2.5
5.0
7.5
Fold Enrichment
Count
3
6
9
12
−1 * log10(PValue)
1.3
1.2
1.1
B
Biology Process
lipid metabolic process
inflammatory response
chemokine−mediated signaling pathway
cell chemotaxis
cell adhesion
5.0
7.5
10.0
12.5
Fold Enrichment
−1 * log10(PValue)
3.5
3.0
2.5
2.0
Count
4
6
8
10
C
Molecular Function
transporter activity
receptor binding
heparin binding
chemokine receptor binding
ATPase activity, coupled to transmembrane movement of substances
0
20
40
60
Fold Enrichment
Count
3
4
5
6
7
−1 * log10(PValue)
3.5
3.0
2.5
2.0
1.5
D
Cell Component
proteinaceous extracellular matrix
integral component of plasma membrane
extracellular space
extracellular region
extracellular exosome
2
4
6
Fold Enrichment
Count
15
20
25
30
−1 * log10(PValue)
6
5
4
3
2

Supplementary Fig 3 The correlation between hub genes and tumor N-stage.

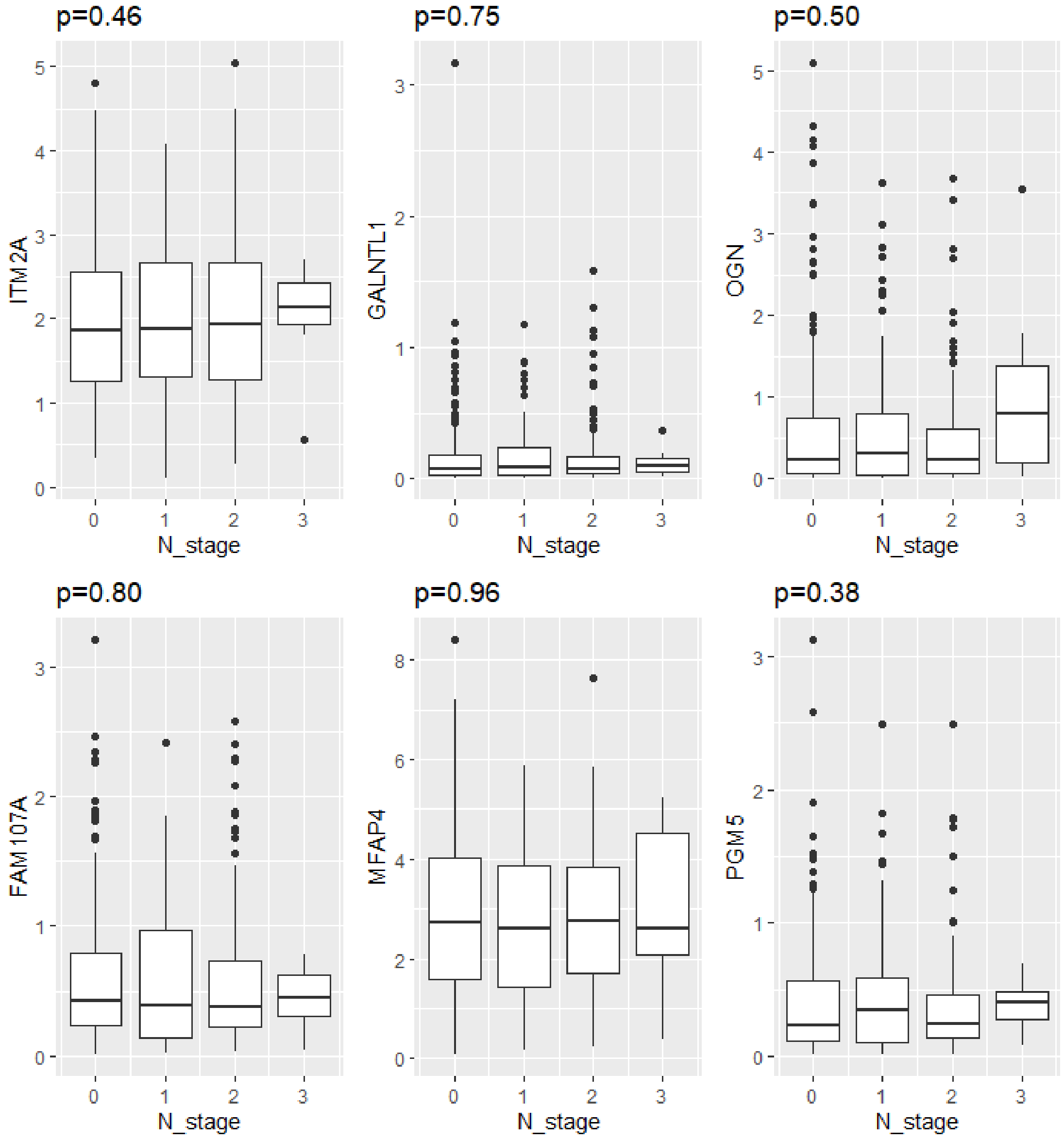

Supplementary Fig 4 ROC curves validate the diagnostic role of hub genes. A: Diagnostic role of hub genes between normal tissue and tumor tissue. B: Diagnostic role of hub genes between tumor tissue and dysplasia tissue.

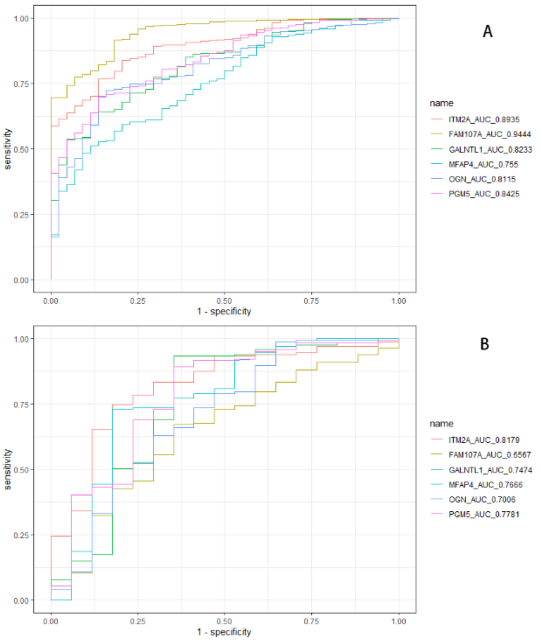

Supplementary Fig 5 Mean methylation level of hub genes between normal and tumor tissue

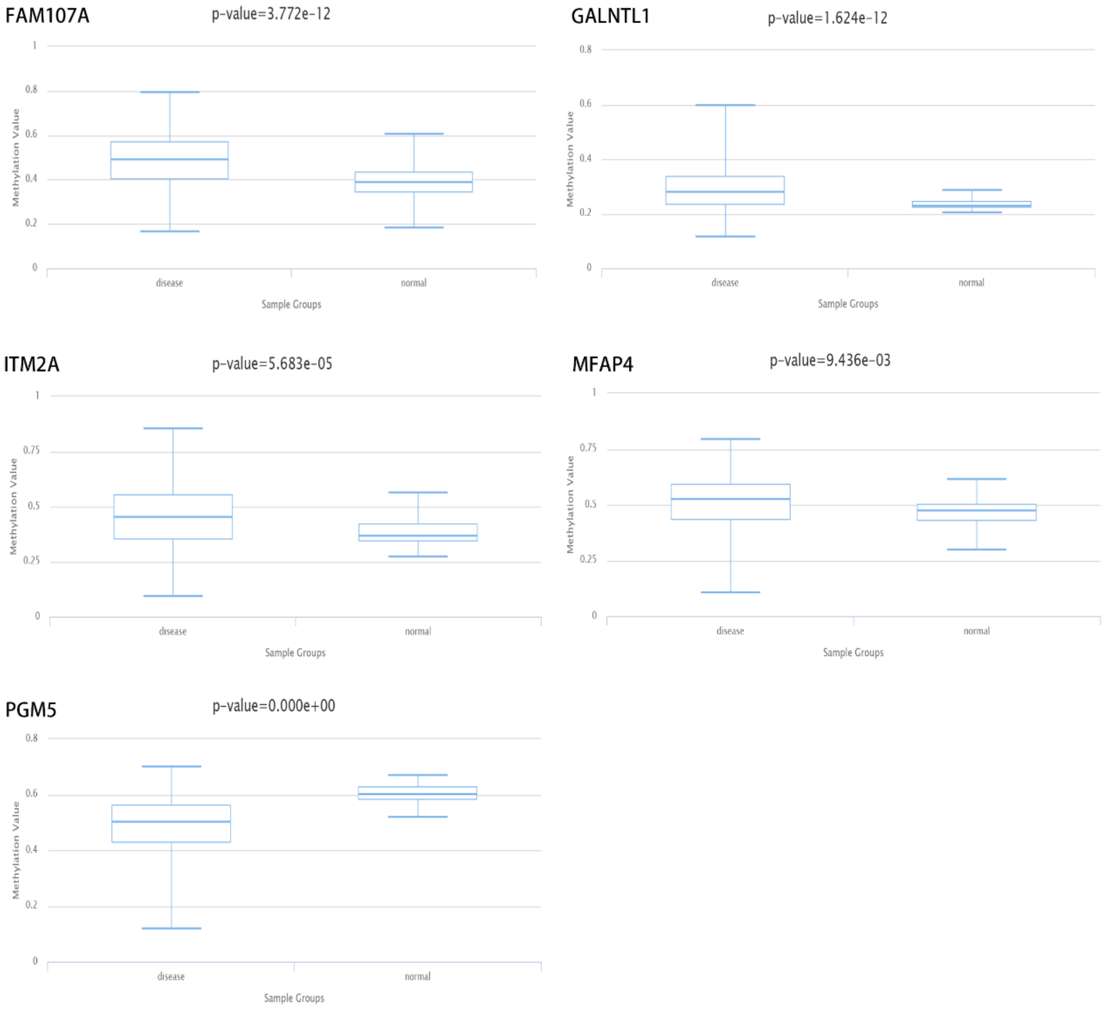

Supplementary Fig 6 The relationship between four hub genes and their methylation site

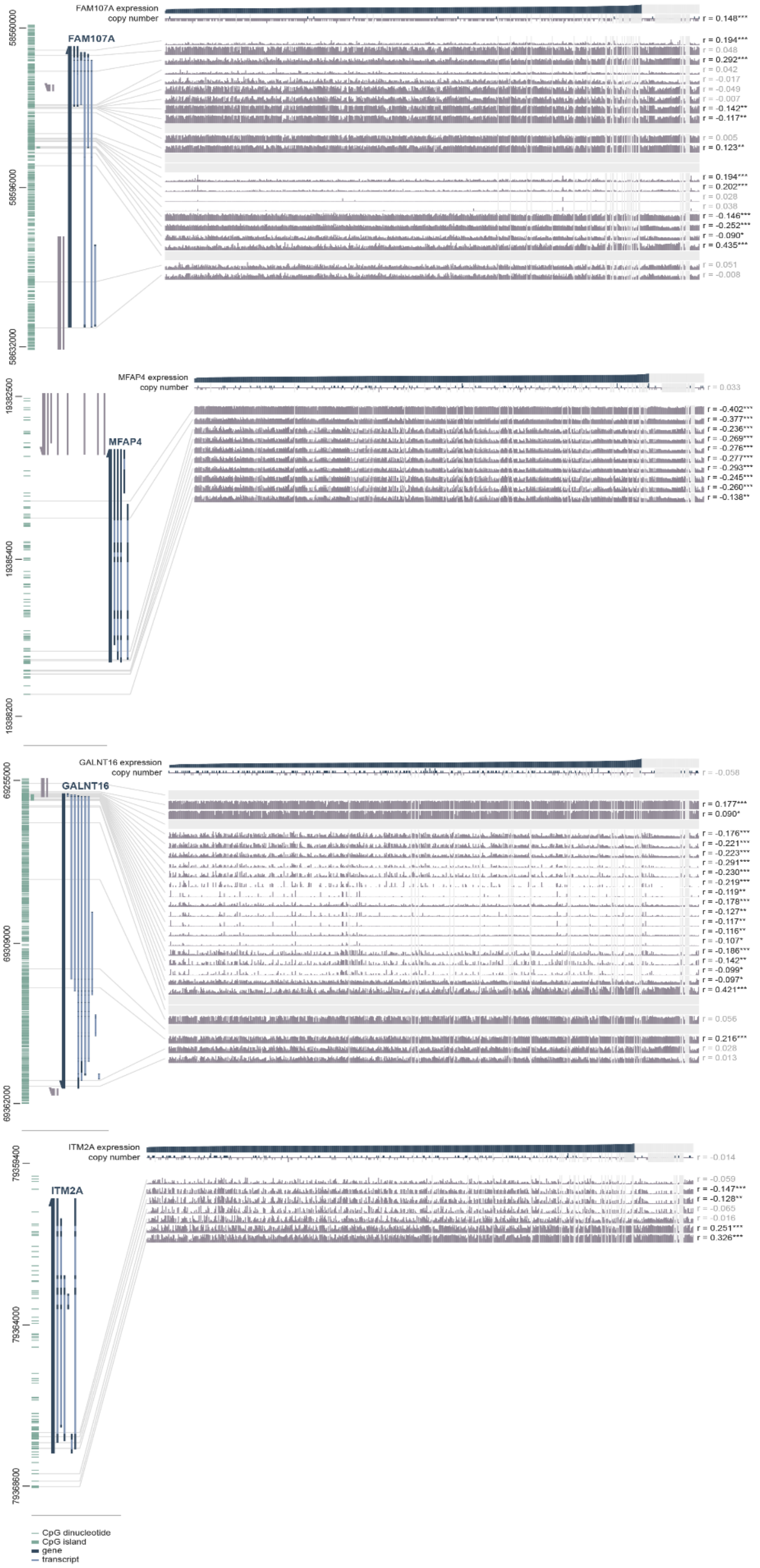

Supplementary Fig 7 GSEA result of hub genes.

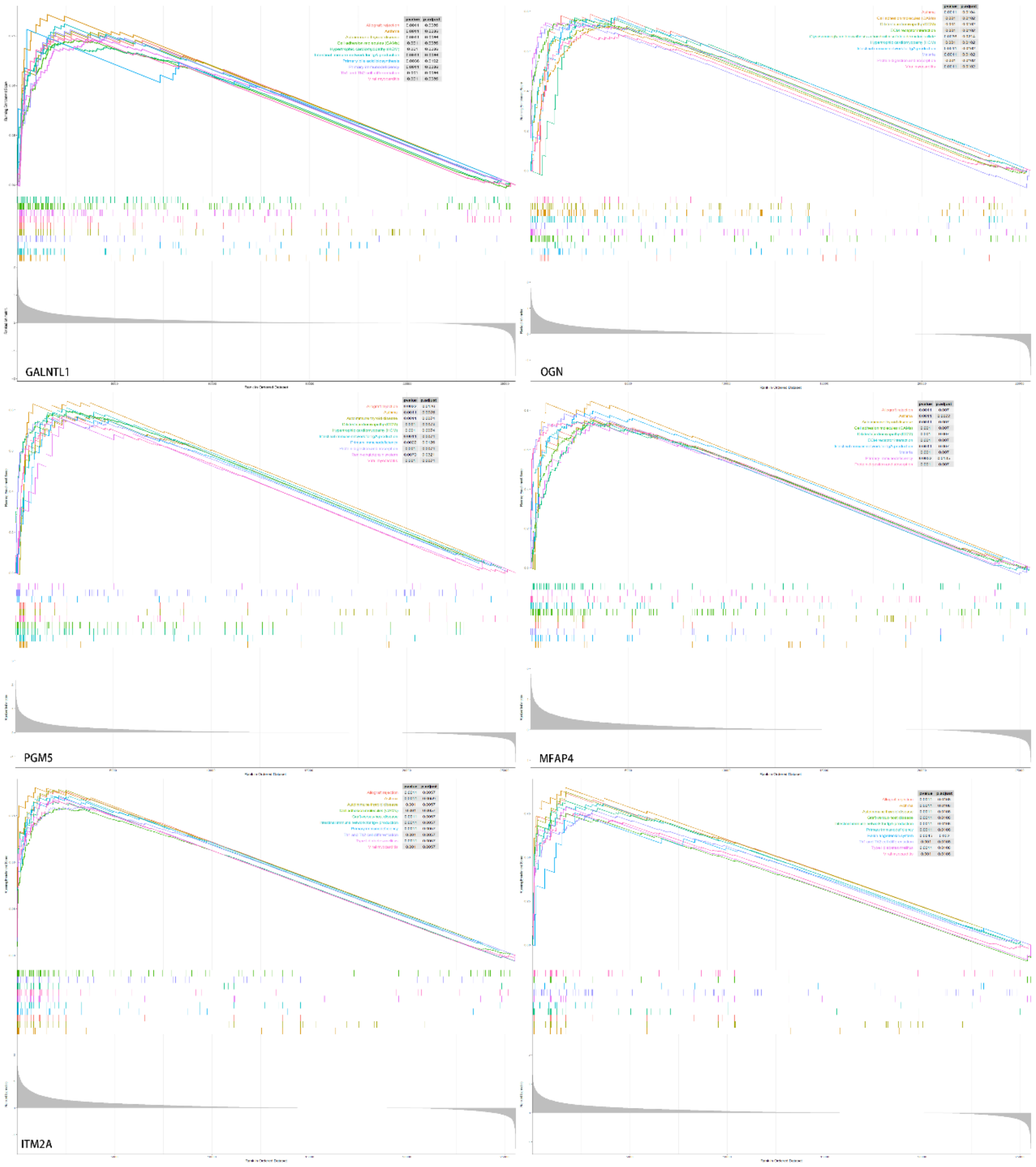
GALNTL1
OGN
PGM5
MFAP4
ITM2A
